# Probing α‑Al_2_O_3_: A Theoretical and Experimental Investigation of Its Optoelectronic, Thermodynamic, and Vibrational Response

**DOI:** 10.1021/acsomega.6c04114

**Published:** 2026-07-16

**Authors:** Edvan Moreira, Leticia de Sousa Costa, Fernando Marques de Oliveira Moucherek, Ediomar Costa Serra, Kariny Alanda Teixeira Costa, Ricardo Gomes, Cleanio da Luz Lima, David Lima Azevedo

**Affiliations:** † Postgraduate Program in Aerospace Engineering and Department of Physics, State University of Maranhão (UEMA), São Luís, Maranhão 65055-970, Brazil; ‡ Postgraduate Program in Aerospace Engineering, State University of Maranhão (UEMA), São Luís, Maranhão 65055-970, Brazil; § State University of Maranhão (UEMA), Caxias, Maranhão 65055-310, Brazil; ∥ Postgraduate Program in Physics, Federal University of Piauí (UFPI), Teresina, Piauí 64049-550, Brazil; ⊥ Institute of Physics, University of Brasilia (UnB), Brasília, Distrito Federal 70910-900, Brazil

## Abstract

This work presents an integrated experimental and first-principles investigation of the structural, electronic, vibrational, optical, and thermodynamic properties of trigonal α-Al_2_O_3_. Experimentally, α-Al_2_O_3_ nanostructures were synthesized and characterized by X-ray diffraction (XRD), UV–vis spectroscopy, and Raman spectroscopy. Rietveld refinement confirmed the predominance of the trigonal corundum structure (space group *R*3̅*c*) with lattice parameters close to experimental reference values. The optical bandgap obtained from UV–vis measurements was approximately 5.6 eV, indicating the influence of nanostructuring and localized states on the optical response. Theoretical calculations were performed within density functional theory (DFT) using both LDA-CAPZ and GGA-PBE exchange-correlation functionals. The calculated electronic structure revealed dominant direct optical transitions near the Γ point with bandgap values between 6.67 and 6.68 eV. Density of states analysis showed that O-2p orbitals dominate the valence band region, while Al 3s/3p states mainly contribute to the conduction bands. Optical absorption and reflectivity spectra exhibited low anisotropy and pronounced ultraviolet absorption characteristics. Vibrational properties calculated through density functional perturbation theory (DFPT) showed good agreement with experimental Raman and infrared spectra. Phonon dispersion calculations revealed the absence of imaginary frequencies, confirming the dynamical stability of trigonal α-Al_2_O_3_. Thermodynamic properties, including enthalpy, entropy, free energy, and heat capacity, were evaluated up to 1000 K, indicating thermal stability and the absence of structural phase transitions within this temperature range. The results establish a consistent correlation between structural stability, electronic structure, vibrational response, and thermodynamic behavior in α-Al_2_O_3_ nanostructures, providing a comprehensive framework for understanding their properties and potential use in optical, catalytic, and high-temperature ceramic applications.

## Graphical abstract

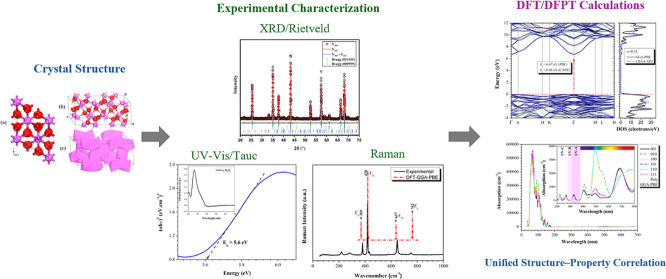

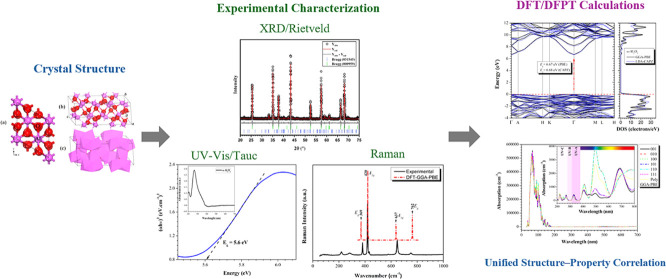

## 1 Introduction

One of the most important ceramic materials for fundamental research is aluminum oxide (Al_2_O_3_) in the alpha phase (α-Al_2_O_3_), a structure called corundum. Its distinctive combination of features, including high hardness, excellent chemical and thermal stability, optical transparency over a wide spectral range, and relevant dielectric properties, makes α-Al_2_O_3_ a model material for both practical applications and fundamental research in surface science and solid-state physics.^1–3^ It is also applied as a surface coating to conventional lithium-based battery materials, improving both their electrochemical performance and long-term cycling capacity.^4,5^ The crystal structure of α-Al_2_O_3_ is trigonal (space group *R*3̅*c*). Alternatively, the structure may be described using a hexagonal primitive cell (rhombohedral-centered hexagonal) containing six units of α-Al_2_O_3_ (12 aluminum-Al atoms and 18 oxygen-O atoms). In this structure, Al^3+^ cations occupy octahedral sites coordinated by six O^2–^ anions, forming alternating layers of oxygen and aluminum along the [0001] direction.^6,7^ First-principles studies using the local density approximation (LDA) and generalized gradient approximation (GGA) have accurately reproduced the experimental lattice parameters (e.g., *a*_hex_ ≈ 4.76 Å, *c*_hex_ ≈ 12.99 Å).^7–10^

Bulk α-Al_2_O_3_ is commonly described as an indirect wide-bandgap insulator in theoretical studies, calculated from LDA/GGA at around 6.0–6.3 eV, which underestimates the experimental optical bandgap value (∼8.8 eV), a well-known discrepancy attributed to the formation of excitons and the limitations of standard density functional theory (DFT) for excited states. However, direct optical transitions near the Γ point may significantly contribute to the optical response, particularly in nanostructured systems and experimental optical absorption measurements.^7,8,11–13^ In the present work, the term “electronic bandgap” refers to the calculated transition energies obtained from DFT calculations, whereas “optical bandgap” refers to the experimentally estimated absorption threshold extracted from UV–vis spectroscopy using the Tauc formalism. The electronic density of states (DOS) of the bulk is dominated by O-2p states in the upper valence band and Al 3s/3p states in the lower conduction band. Surface formation introduces localized electronic states within the bulk bandgap and modifies the density of states near the Fermi level.^8^

In heterogeneous catalysis and surface functionalization, α-Al_2_O_3_ serves as an inert support for metal nanoparticles or as a direct adsorbent for small molecules. To elucidate the interaction mechanisms, adsorption energies, and changes in vibrational properties, the adsorption of small molecules such as H_2_O, HCl, CH_3_OH, and especially carbon monoxide (CO) on the (0001) surface was studied.^8,14^ The CO molecule serves as a versatile molecular probe, with shifts in the stretching vibrational frequency (νCO) and variations in adsorption energy offering insights into the characteristics of the adsorption site, charge transfer, and structural relaxations caused by surface coverage.^8^ DFT studies on α-Al_2_O_3_ (0001) show that CO is adsorbed molecularly via a carbon atom on an aluminum surface site, with a weak adsorption energy (∼0.4–0.5 eV), causing a blue shift in the νCO frequency (30–56 cm^–1^) relative to the gas phase.^8,15^

Another important research direction involves the synthesis and characterization of thin films and nanoparticles of α-Al_2_O_3_. The pure alpha phase with a hexagonal structure can be obtained using the sol–gel method followed by high-temperature calcination (*T* ≥ 1100 °C), as confirmed by X-ray diffraction (XRD) and Raman spectroscopy.^16,17^ The morphology (pores, large grains) and crystallite size (30–40 nm) are sensitive to calcination temperature. Optical measurements (UV–vis) on sol–gel synthesized nanoparticles indicate direct bandgaps (∼3.8–4.5 eV) smaller than the bulk value, which is attributed to surface defect states or vacancies that generate levels within the bandgaps.^7,16^ IR spectroscopy on thin films of α-Al_2_O_3_ grown by oxidation shows excellent agreement with ab initio simulations of the vibrational modes (transverse optical—TO and longitudinal optical—LO) of the bulk material. However, it also reveals additional modes that can be attributed to residual phases of transition alumina, interface modes, or impurities such as hydroxyls and adsorbed water.^17^ Compared with conventional bulk α-Al_2_O_3_, nanostructured α-Al_2_O_3_ can exhibit additional surface-related effects due to its higher surface-to-volume ratio, including surface disorder, localized electronic states, and modified optical or vibrational responses. Therefore, correlating theoretical bulk-like calculations with experimental nanostructured samples is useful for understanding how nanoscale effects may influence the structure–property relationships of α-Al_2_O_3_.

This work provides a unified experimental–theoretical correlation between vibrational stability, thermodynamic behavior, and anisotropic optical response in α-Al_2_O_3_ nanostructures, which remains insufficiently explored in the literature. First-principles calculations based on density functional theory (DFT) were employed to investigate its structural, optoelectronic, thermodynamic, and vibrational properties, including phonon, infrared (IR), and Raman spectra. These theoretical results are complemented by experimental characterization using X-ray diffraction (XRD), UV–vis spectroscopy, and Raman spectroscopy. Such a combined approach enables a deeper understanding of α-Al_2_O_3_, both in its ideal crystalline form and in synthesized nanostructures (Figure 1) and provides valuable insights for the design of alumina-based materials with optimized performance in catalytic systems, protective coatings, and electronic devices. Despite the extensive literature on α-Al_2_O_3_, most studies focus on isolated properties such as electronic structure or vibrational behavior. Although several structural, optical, vibrational, and electronic properties of α-Al_2_O_3_ have been reported previously, these investigations are typically presented independently, focusing on isolated aspects of the material. In contrast, the present work combines experimental characterization (XRD, UV–vis, Raman, and IR spectroscopy) with first-principles DFT/DFPT calculations and thermodynamic analysis within a single framework. This integrated approach enables a direct comparison between theoretical predictions and experimental observations and provides a more comprehensive understanding of the relationships among structural, electronic, vibrational, optical, and thermodynamic properties in nanostructured α-Al_2_O_3_. By combining XRD, UV–vis, Raman spectroscopy, DFT, DFPT, phonon dispersion, and thermodynamic calculations within a unified framework, the work seeks to provide a more comprehensive understanding of how these properties are interconnected in α-Al_2_O_3_ nanostructures. Rather than introducing a previously unreported individual property, the novelty of this work lies in the systematic correlation of complementary experimental and theoretical data sets and in the unified interpretation of the material behavior across multiple physical domains.

**Figure 1:**
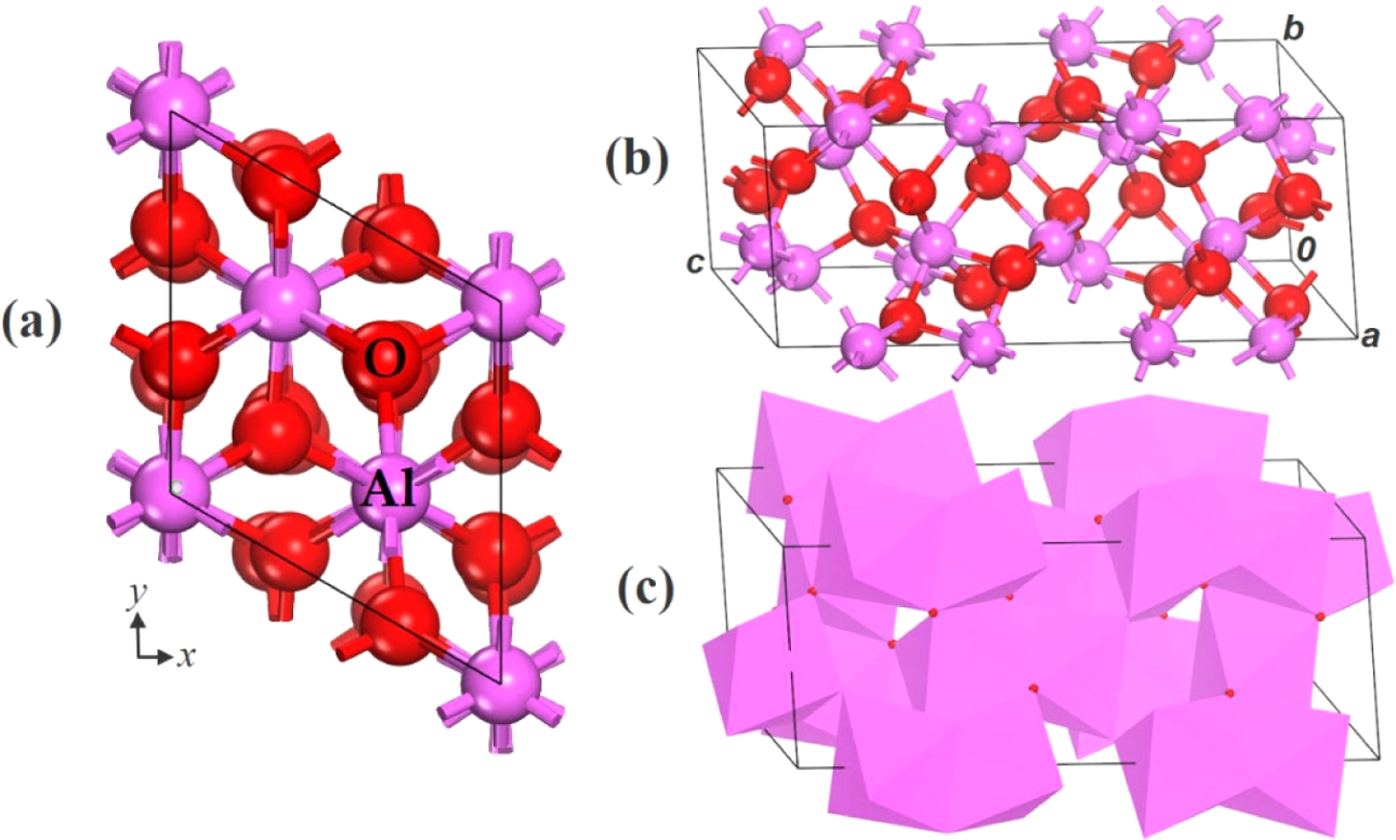
The structure of trigonal α-Al_2_O_3_. (a,b) Different views of the primitive cell and atomic labels; and (c) polyhedral form. The structure consist of aluminum-Al (pink ball), and oxygen-O (red ball). (For interpretation of the references to colors in this figure legend, the reader is referred to the web version of this paper.)

## 2 Methodology

### 2.1 Experimental Section

The analyses were performed on a high-purity (99.98%) aluminum oxide (Al_2_O_3_) sample in powder form. The sample was characterized using powder X-ray diffraction (PXRD) at ambient temperature. All experimental characterizations (XRD, Raman, and UV–vis) were conducted under standard ambient laboratory conditions, at a temperature of approximately 23 ± 2 °C and relative humidity below 60%. Figure 2 presents the resulting PXRD pattern plotted together with the refined pattern from Rietveld analysis.

**Figure 2:**
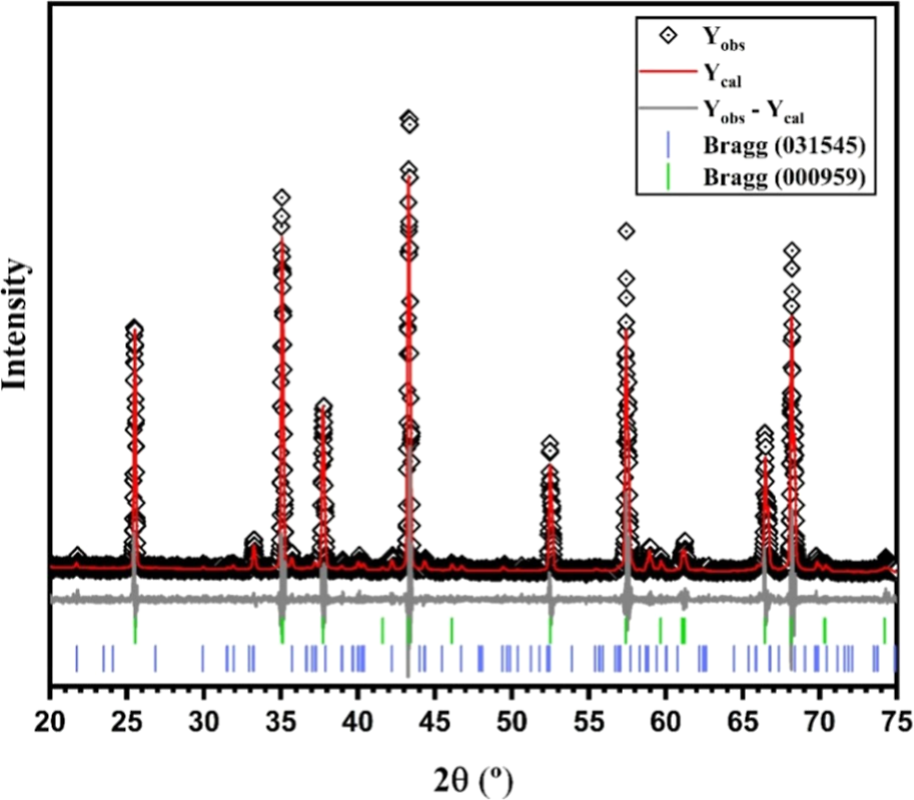
X-ray diffraction patterns observed (red circle) and calculated by the Rietveld method (black line) of α-Al_2_O_3_ (*R*_wp_ = 17.32% and GOF = 1.23). (For interpretation of the references to colors in this figure legend, the reader is referred to the web version of this paper.)

The powder X-ray diffraction (PXRD) analysis was performed with a Bruker D8 discover diffractometer equipped with a LynxEye-XE linear detector and a Cu Kα radiation source (λ = 1.5418 Å). The X-ray tube was operated at 40 kV and 40 mA. Data collection spanned a 2θ range from 20° to 75°, using a step size of 0.02° and a counting time of 0.5 s per step.

Structural analysis was performed to determine the structural parameters of the samples through the Rietveld method,^18^ with calculations carried out using GSAS-II software.^19^

Aluminum oxide (Al_2_O_3_) was also characterized by Raman spectroscopy to verify phase formation, identify the molecular vibration modes of the phases present, and compare the Raman data obtained by computational simulation. The tests were performed using a LabRAM HR evolution Raman spectrometer (HORIBA) with a λ = 532 nm laser line, 50× focusing lens, measurements with 3 accumulations of 10 s each at 2 different points on the sample, covering the spectral range from 50 to 1100 cm^–1^. The resolution applied was 2.0 cm^–1^, with an excitation power of 1.0 mW.

For the UV–vis spectroscopy experiment, a Shimadzu UV-2600 visible ultraviolet spectrophotometer was used to analyze samples and acquire the spectrum of absorbance. The scanned spectral range was from 200 to 700 nm with a medium scan speed.

### 2.2 Computational Methods

The physical characteristics of the system were modeled computationally using density functional theory (DFT) formalism,^20,21^ implemented through the CASTEP code.^22^ The implementation of the local density approximation (LDA) leveraged the exchange-correlation functional parametrized by Perdew and Cerpeley (CAPZ),^23,24^ while the generalized gradient approximation (GGA) utilized the Perdew–Burke–Ernzerhof (PBE) parameters.^25^ In both approaches, electron–ion interactions were treated with pseudopotentials. Specifically, the LDA-CAPZ calculations employed Vanderbilt ultrasoft pseudopotentials^26^ and for the GGA calculations, norm-conserved pseudopotentials^27^ were applied, which enhance computational efficiency by permitting a lower plane-wave kinetic energy cutoff without compromising the precision of derived electronic, optical, vibrational, and thermodynamic observables. Although different pseudopotential libraries were employed for the LDA-CAPZ and GGA-PBE calculations, the purpose of the comparison was to evaluate the consistency of the predicted trends rather than to establish a strict quantitative benchmark between exchange-correlation functionals. Therefore, the results are discussed primarily in terms of qualitative agreement and overall physical behavior. For the present study, a plane-wave basis set with a cutoff energy of 830 eV was adopted, resulting in well-converged numerical outcomes.

The valence electronic configurations for the constituent elements were defined as 3s^2^3p^1^ for aluminum (Al) and 2s^2^2p^4^ for oxygen (O). A Monkhorst–Pack sampling^28^ of the Brillouin zone was conducted using a 4 × 4 × 2 *k*-point grid for reciprocal space integration. To establish a reliable structural model and obtain the ground-state configuration, the atomic coordinates within the nanostructure were fully relaxed. This geometry optimization procedure was executed consistently under both the LDA-CAPZ and GGA-PBE approximations, maintaining a plane-wave energy cutoff of 830 eV to ensure well-converged total energies.

To systematically converge the system to its ground-state minimum energy configuration, a stringent set of criteria was enforced during the iterative self-consistent field (SCF) cycles. The optimization was considered complete when sequential steps simultaneously satisfied the following thresholds: a tolerance in total energy change of 0.5 × 10^–5^ eV/atom, a maximum residual force on any ion below 0.01 eV/Å, an internal system pressure under 0.02 GPa, and a maximum ionic displacement between steps less than 0.5 × 10^–3^ Å. Geometry relaxation to the fundamental energy state was driven by the Broyden–Fletcher–Goldfarb–Shanno (BFGS) algorithm.^29^ Within this scheme, atomic-level convergence was rigorously controlled by tolerances of 0.5 × 10^–6^ eV for the total energy per atom and 0.2083 × 10^–6^ eV for Eigen-energy, with a convergence window spanning three consecutive SCF cycles. All calculations were performed using a plane-wave basis set with a kinetic energy cutoff fixed at 830 eV. Consistency in the quality of the electronic structure description was ensured by dynamically accounting for changes in the primitive cell volume throughout the optimization procedure.

Upon establishing the fully optimized primitive cell within the GGA framework, its vibrational characteristics were investigated, using only norm-conserved pseudopotential. The infrared (IR) and Raman scattering spectra were computed, along with the frequencies of their corresponding active phonon modes, following a methodology consistent with prior work.^30–34^ Furthermore, thermodynamic and vibrational properties were derived within the harmonic approximation by applying density functional perturbation theory (DFPT), as implemented through a linear response formalism.^35,36^

The geometric optimization parameters used in this stage of the study were more rigorous compared to those applied for the LDA approximation. The iterative relaxation procedure was governed by stringent convergence thresholds. Structural optimization was considered complete when the following conditions were met simultaneously: a variation in total energy per atom below 5.0 × 10^–6^ eV/atom, maximum ionic force smaller than 0.01 eV/Å, maximum ionic displacements between steps less than 5.0 × 10^–4^ Å, and internal stress components below 0.02 GPa. For the electronic structure minimization within each ionic step, convergence required a change in total energy per atom smaller than 0.1 × 10^–9^ eV, and a shift in Eigen-energy below 0.1 × 10^–8^ eV.

The primitive cell of aluminum trioxide (Al_2_O_3_) nanostructure is shown in perspective in Figure 1a,b, and its polyhedral form c. The primitive cell for Al_2_O_3_ is alpha (α) trigonal (trigonal α-Al_2_O_3_) and, contains 12 aluminum (Al) atoms and 18 oxygen (O) atoms. The space group for α-Al_2_O_3_ is *R*3̅*c* (#167).

## 3 Results and Discussion

### 3.1 X-ray Diffraction Analysis

The X-ray diffraction pattern of α-Al_2_O_3_ reveals a trigonal crystal structure, conforming to the standard model (ICSD no. 14332), with the lattice parameters: *a* = *b* = 4.75(9) Å, and *c* = 12.99(27) Å, V = 254.38(8) Å^3^, α = β = 90°, and γ = 120°. X-ray diffraction showed peaks characteristic of the alpha (α) phase of Al_2_O_3_, which is consistent with the literature.^37–39^ The diffraction peaks observed at 25.59°, 35.07°, 37.73°, 43.36°, 52.47°, 57.44°, 66.43°, and 68.20° of synthesized α-Al_2_O_3_ nanoparticles are attributed to the (012), (104), (110), (113), (024), (116), (214), and (300) lattices planes, respectively, with the preferred crystallographic direction being the (113) plane, followed by (104), (116), and (300), with a significant number of other planes. These lattice planes provide evidence for the formation of the trigonal phase of Al_2_O_3_ ^39^ and are illustrated in Figure 2.

The X-ray diffraction analysis confirms that the crystalline structure of the sample is predominantly composed of trigonal α-Al_2_O_3_, crystallizing in the corundum structure with space group *R*3̅*c*. The diffraction peaks are sharp and well-defined, indicating good crystallinity and structural ordering. The Rietveld refinement was performed considering the trigonal structural model, and the calculated diffraction profile shows excellent agreement with the experimental data, as evidenced by the strong overlap between observed (*Y*_obs._) and calculated (*Y*_calc._) intensities across the entire 2θ range. The refinement converged with the following reliability factors GOF = 1.23 and *R*_wp_ = 17.32, shown in Figure 2. The difference curve (*Y*_obs._ – *Y*_calc._) exhibits low residual intensity and no systematic deviations, reinforcing the reliability of the refined structural parameters. The obtained GOF value, close to unity, indicates a high-quality refinement and confirms that the trigonal structural model adequately describes the experimental diffraction pattern. The weighted profile factor (*R*_wp_) is within the acceptable range for oxide ceramic systems, further validating the robustness of the refinement.

Although minor low-angle reflections are observed, their intensity is negligible compared to the dominant trigonal phase, and they do not affect the structural integrity or the refinement quality of the α-Al_2_O_3_ phase. Overall, the refinement results confirm that the trigonal α-Al_2_O_3_ phase is the principal crystalline structure in the sample, providing a solid structural basis for the subsequent electronic structure calculations performed within the DFT framework. Quantitative phase analysis revealed that the sample consists of approximately 89.3% alpha phase (trigonal), and 10.7%, from a secondary hydrated hexagonal phase (space group *P*6_3_/*mmc*), *a* = *b* = 5.59(8) Å, *c* = 22.61(15) Å, *V* = 612.97(10) Å^3^, α = β = 90°, and γ = 120°, in agreement with ICSD no. 000959. Although the secondary phase content is limited, its possible influence on the optical absorption behavior and vibrational features cannot be completely excluded.

Table 1 summarizes the calculated structural parameters of α-Al_2_O_3_, as derived from the LDA-CAPZ and GGA-PBE approaches, and compared with other studies.^7,9,40,41^ The corresponding experimental values are also listed for direct comparison. The parameters obtained with LDA and GGA approaches underestimate (Δ) the experimental values, 1.05 and 2.94%, taking into account parameters *a*, *b*, and *c*, while volume *V* does not exceed 8.23% (ΔExp. – PBE). Fractionary atomic coordinates for α-Al_2_O_3_ are shown in Table 2.

**Table 1:** Lattice Parameters for Trigonal (Rhombohedral-Centered Hexagonal) Al_2_O_3_ Crystal Calculated Within the GGA-PBE (Norm-Conserving Potential) and LDA-CAPZ Approaches, as Well as the Experimental Data (Exp.)^a^

Al_2_O_3_	*a* = *b* (Å)	*c* (Å)	*V* (Å^3^)	α = β (°)	γ (°)
Exp.	4.75	12.99	254.38	90.0	120.0
GGA-PBE	4.61	12.64	233.44	90.0	120.0
Δ (Exp. – PBE)	2.94%	2.69%	8.23%	0.0	0.0
LDA-CAPZ	4.70	12.81	245.51	90.0	120.0
Δ (Exp. – CAPZ)	1.05%	1.38%	3.48%	0.0	0.0
Exp.^40^	4.756	12.982	254.338	90.0	120.0
r++SCAN^7^	4.781	13.022	257.787	90.0	120.0
LDA^41^	4.709	12.846	246.740	90.0	120.0
LDA^9^	4.7901	12.9746	-	90.0	120.0
GGA-PBE^41^	4.784	13.068	259.072	90.0	120.0

a Lengths (*a*, *b*, *c*) are in Å, volumes (V) in Å^3^, and angles (α, β, γ) in degrees.

**Table 2:** Internal Coordinates for Trigonal Al_2_O_3_ According with Experimental Data^a^

element	internal coordinates		
	*u*	*v*	*w*
O_1_	0.308373	0.000000	0.250000
O_2_	0.975040	0.333333	0.583333
O_3_	0.641706	0.666667	0.916667
O_4_	0.000000	0.308373	0.250000
O_5_	0.666667	0.641706	0.583333
O_6_	0.333333	0.975040	0.916667
O_7_	–0.308373	–0.308373	0.250000
O_8_	0.358294	0.024960	0.583333
O_9_	0.024960	0.358294	0.916667
O_10_	–0.308373	0.000000	–0.250000
O_11_	0.358294	0.333333	0.083333
O_12_	0.024960	0.666667	0.416667
O_13_	0.000000	–0.308373	–0.250000
O_14_	0.666667	0.024960	0.083333
O_15_	0.333333	0.358294	0.416667
O_16_	0.308373	0.308373	–0.250000
O_17_	0.975040	0.641706	0.083333
O_18_	0.641706	0.975040	0.416667
Al_1_	0.000000	0.000000	0.353005
Al_2_	0.666667	0.333333	0.686339
Al_3_	0.333333	0.666667	1.019672
Al_4_	0.000000	0.000000	0.146995
Al_5_	0.666667	0.333333	0.480328
Al_6_	0.333333	0.666667	0.813661
Al_7_	0.000000	0.000000	–0.353005
Al_8_	0.666667	0.333333	–0.019672
Al_9_	0.333333	0.666667	0.313661
Al_10_	0.000000	0.000000	0.853005
Al_11_	0.666667	0.333333	1.186339
Al_12_	0.333333	0.666667	1.519672

a The coordinates (*u*, *v*, *w*) are measured relative to the *a*, *b*, and *c* lattice parameters of the primitive cell, respectively.

Table 2 compiles the XRD diffraction peak positions, with the corresponding crystallographic data derived from Rietveld refinement analysis. Then, based on X-ray diffraction (XRD) measurements, the lattice parameters and crystallographic data obtained allowed the determination of the α-Al_2_O_3_ crystallographic information file (CIF), which, in turn, enabled the theoretical calculation of the nanostructure’s physical properties.

### 3.2 Ultraviolet–Visible Absorption Spectroscopy

The optical characterization of α-Al_2_O_3_ was performed via UV–vis spectroscopy across the 200–700 nm wavelength range (Figure 3 inset). The spectra reveal a shift in the fundamental absorption edge. This shift can be interpreted by analyzing the optical bandgap of the nanostructure through application of the Tauc relation,^42,43^ as follows

$${\left(\alpha h\nu \right)}^{n}=A\left(h\nu -E_{g}\right)$$

**Figure 3:**
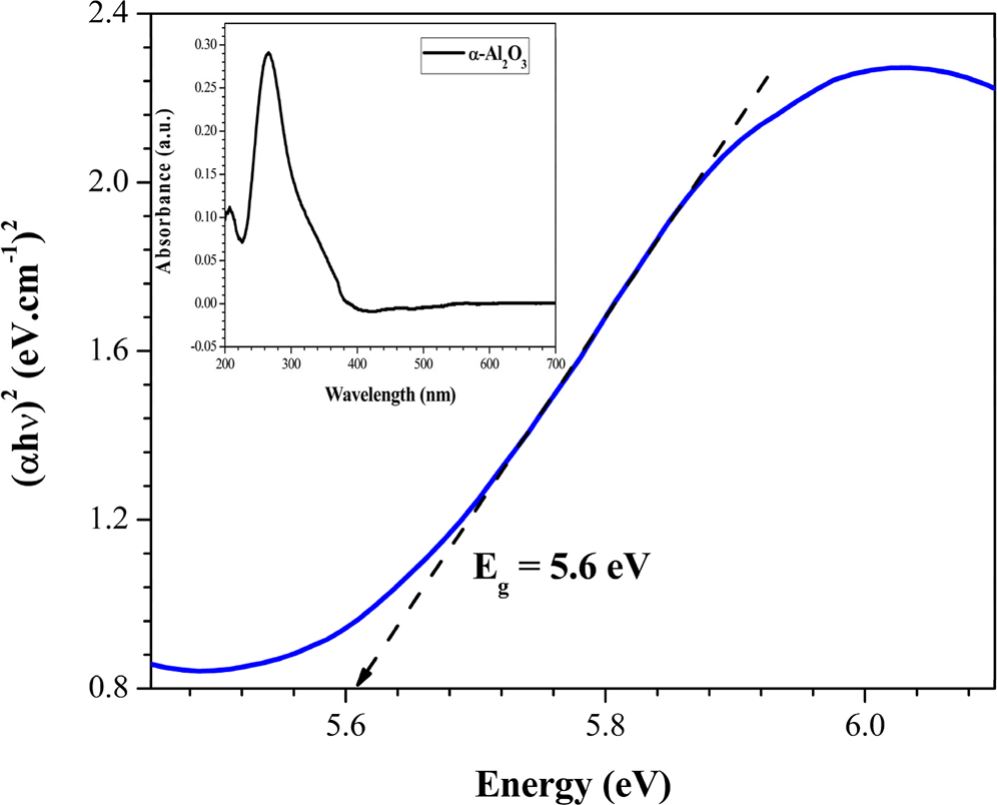
Plot of reflectance of [*F*(*R*) *h*ν]^2^ versus energy (eV) for α-Al_2_O_3_, with an inset showing the absorption spectrum of α-Al_2_O_3_.

In this expression, α is the absorption coefficient, *h*ν is the photon energy, and *A* is a proportionality constant. The exponent *n* is 2 for a direct bandgap and 1/2 for an indirect bandgap, characterizing the nature of the optical transition, while *E*_g_ denotes the derived optical bandgap energy.

Figure 3 presents a Tauc plot of (α*h*ν)^2^ versus photon energy. The optical bandgap for α-Al_2_O_3_ was estimated at (*E*_g_ = 5.6 eV) by extrapolating the linear region of this plot to the *x*-axis (less than 6.2 eV^16^). The experimental optical bandgap (∼5.6 eV) is lower than both the calculated electronic bandgap values and the optical gaps commonly reported for bulk α-Al_2_O_3_. This discrepancy may arise from the fact that the optical absorption onset in nanostructured alumina systems can be influenced by multiple factors, including finite-size effects, possible localized electronic states, surface disorder, residual hydrated secondary phases identified by Rietveld refinement, and broadening effects associated with experimental absorption measurements. In addition, the Tauc analysis employed in this work describes the dominant optical transition behavior observed experimentally rather than the ideal bulk electronic band structure. Therefore, the reduced optical bandgap should not be interpreted exclusively as evidence of intrinsic defect formation or as a direct representation of the intrinsic bulk electronic bandgap of ideal α-Al_2_O_3_. However, the present experimental and theoretical results do not allow a definitive identification of the specific defect types, concentrations, or their individual contributions to the observed optical absorption behavior. The Tauc analysis was performed considering dominant direct optical transitions (Γ → Γ), which are commonly employed to describe the optical absorption onset in nanostructured alumina systems. This approach was adopted to analyze the experimental absorption edge observed in the UV–vis spectra rather than to categorically define α-Al_2_O_3_ as a purely direct-gap material. In literature, experimental studies using UV–vis spectroscopy show that the bandgap of α-Al_2_O_3_ are presented in two energy ranges: 0.94–3.5 eV and 4.5–8.8 eV,^16,44,45^ confirming also the plausibility of the theoretical result found.^7,8,13,16,41^ The reduced experimental bandgap compared to theoretical values highlights the influence of defects and surface states, which are particularly relevant for tuning optical properties in nanostructured materials. In addition, the minor hydrated secondary phase identified by Rietveld refinement may contribute to localized absorption features extending beyond the ideal ultraviolet absorption region expected for bulk α-Al_2_O_3_.

The Tauc analysis employed in this work was performed considering dominant direct optical transitions (Γ → Γ), since the experimental absorption edge exhibited a more linear behavior under the direct-transition formalism. This approach was adopted to describe the experimentally observed optical absorption onset in the synthesized nanostructured alumina system rather than to categorically redefine the intrinsic bulk electronic band structure of α-Al_2_O_3_. The absorption features below 200 nm are consistent with high-energy interband transitions associated with the wide-bandgap nature of α-Al_2_O_3_. In contrast, the features observed in the visible range (∼400–600 nm) are not directly predicted by the ideal bulk electronic band structure, which shows no intrinsic electronic states within the gap region. Therefore, these visible-range features are more appropriately interpreted as extrinsic or sample-dependent optical contributions, possibly related to localized states, surface disorder, nanostructuring effects, and the residual hydrated secondary phase identified by Rietveld refinement.

### 3.3 Band Structure and Partial Density of States

This section presentes the Kohn–Sham electronic band structure^21^ that describes the dependence of energies *E* on the wave vector *k* in the first Brillouin zone.

Figure 4 shows the energy bands for α-Al_2_O_3_, also calculated using the GGA-PBE (solid black lines) and LDA-CAPZ (dashed blue lines) exchange–correlation functionals, containing about 42 valence bands between −5.0 and 0.0 eV and, a set of approximately 24 conduction bands from 6.0 to 13.0 eV.

**Figure 4:**
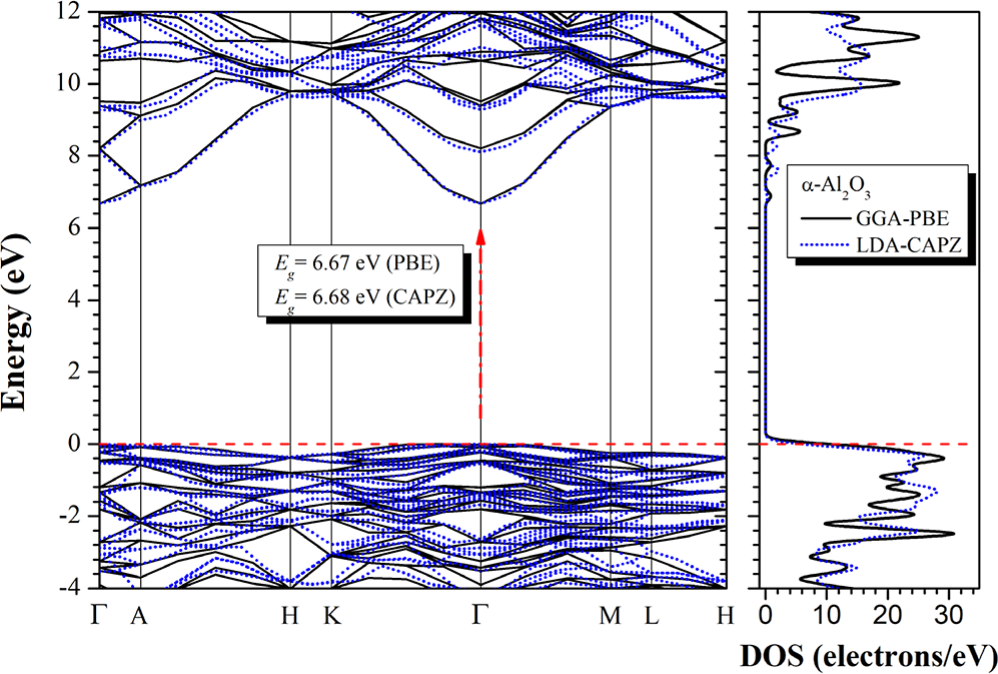
(Left) Electronic band structure near the Fermi level (chosen to be zero) and (right) total density of states (DOS) of trigonal α-Al_2_O_3_ calculated using the GGA-PBE (solid black lines) and LDA-CAPZ (dashed blue lines) exchange-correlation functionals. (For interpretation of the references to colors in this figure legend, the reader is referred to the web version of this paper.)

The Brillouin zone of the α-Al_2_O_3_ structure was delineated by selecting straight-line segments connecting points of high symmetry. The points of high symmetry (Γ-A-H-K-Γ-M-L-H) were chosen by the same calculation scheme employed for the structural analysis: Γ(0.000; 0.000; 0.000), A(0.000; 0.000; 0.500), H(−0.333; 0.667; 0.500), K(−0.333; 0.667; 0.00), M(0.000; 0.500; 0.000), and L(0.000; 0.500; 0.500).

Figure 4 displays the calculated electronic band structure. The results from the LDA-CAPZ functionals (dashed blue lines) show a direct bandgap of 6.68 eV (6.3 eV,^13^ 6.594 eV,^38^ 6.29 eV^46^), while those from the GGA-PBE functionals (solid black lines) indicate a direct gap of 6.67 eV (6.045 eV^41^). In both calculations, the top of the valence band, set as the Fermi level (at 0.0 eV), and the bottom of the conduction band are located at the same high-symmetry point Γ in reciprocal space, confirming the direct bandgap for this structure (Γ → Γ).^41^ The approximations intrinsic to the DFT formalism mean that the bandgap values it provides are estimates, and conventional LDA and GGA exchange–correlation functionals are well-known to systematically underestimate semiconductor and insulator bandgaps due to the exchange-correlation approximation. In the present work, the calculated electronic transition energies are higher than the experimental optical bandgap obtained from UV–vis measurements. This difference may arise because the experimentally estimated optical absorption onset in nanostructured alumina systems does not necessarily correspond to the intrinsic bulk electronic bandgap.^7,41^ The reduction in the optical bandgap observed experimentally, compared to the theoretical bulk value, can be attributed to surface defects and localized states that are more prominent at the nanoscale. These defect-induced states can significantly influence charge transport and optical absorption, making α-Al_2_O_3_ nanostructures particularly relevant for applications requiring tunable electronic properties. In addition to the band structure, the total density of states (DOS) was analyzed to provide further insight into the electronic structure of α-Al_2_O_3_. As shown in Figure 4, both exchange-correlation functionals predict a wide energy gap separating the occupied and unoccupied states. The DOS profile corroborates the band structure results and confirms the insulating nature of α-Al_2_O_3_, with the gap region remaining essentially free of electronic states. This electronic configuration directly influences the optical absorption behavior, particularly in the UV region, reinforcing the suitability of α-Al_2_O_3_ for optoelectronic applications.

Figure 5 shows the partial density of states (PDOS) profiles for the α-Al_2_O_3_ structure, showing the relationship between energy (eV) and the chemical elements (a) aluminum (Al) and (b) oxygen (O), as well as the respective atomic orbitals (s and p), using DFT calculations employing the GGA-PBE approximation, close to the Fermi level (*E* = 0 eV).

**Figure 5:**
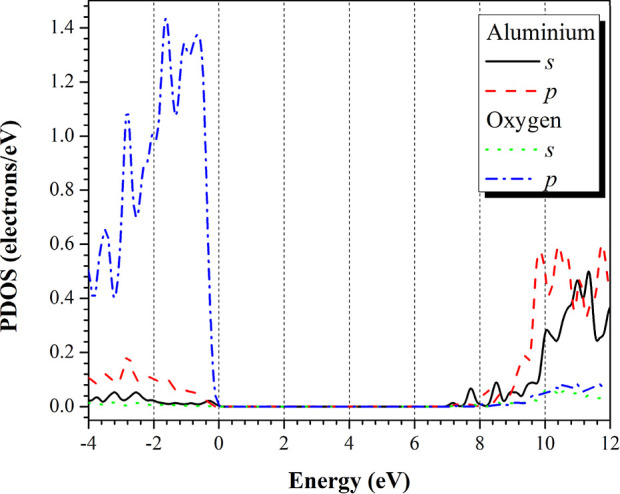
GGA-PBE partial density of states (PDOS) of trigonal α-Al_2_O_3_ per atom of aluminum (Al) and oxygen (O), and per orbital type. The Fermi level is indicated as the line at *E* = 0 eV. (For interpretation of the references to colors in this figure legend, the reader is referred to the web version of this paper.)

The results reveal that the electronic structure of α-Al_2_O_3_ tends to be influenced more by the 2p^4^ and 3p^1^ orbitals of oxygen (O) and aluminum (Al), respectively, in the regions below and above the Fermi level, between −4.0 and 12.0 eV, in accordance with other studies.^7,8,46^ However, in the region just below the Fermi level, the oxygen 2p^4^ orbital is predominant, essentially defining the formation of the valence bands, while in the region above the Fermi level, the aluminum 3p^1^ orbital predominates, with considerable influence from the 3s^2^ orbital between 7.0 and 8.0 eV, acting mainly in the formation of the conduction bands. However, it can be observed that, in the vicinity of the Fermi level, the effects associated with the O-2p orbitals are more relevant, resulting in higher energy bandgaps.

### 3.4 Optical Properties

#### 3.4.1 Optical Absorption

Figure 6 depicts the optical absorption as a function of wavelength (in nm) for the α-Al_2_O_3_ nanostructure from the GGAPBE functional. The absorption spectrum for nanostructures satisfies the Planck–Einstein relationship, *E* = *h*ν, where *h* is Planck’s constant, and ν is the frequency of the radiation, here in wavelength (nm). The horizontal axis represents the wavelength (λ) in nanometers (nm), ranging from 0 to 700 nm, while the vertical axis represents the absorption coefficient in units of cm^–1^, and shows how the analyzed material absorb radiation in the UV (UV-A, UV-B, and UV-C) and visible regions (inset 400–700 nm). The specified curves ([001], [010], [100], [101], [110], [111]) correspond to different crystallographic directions of polarization of incident light (Miller’s indices) and the average over all directions (Poly).

**Figure 6:**
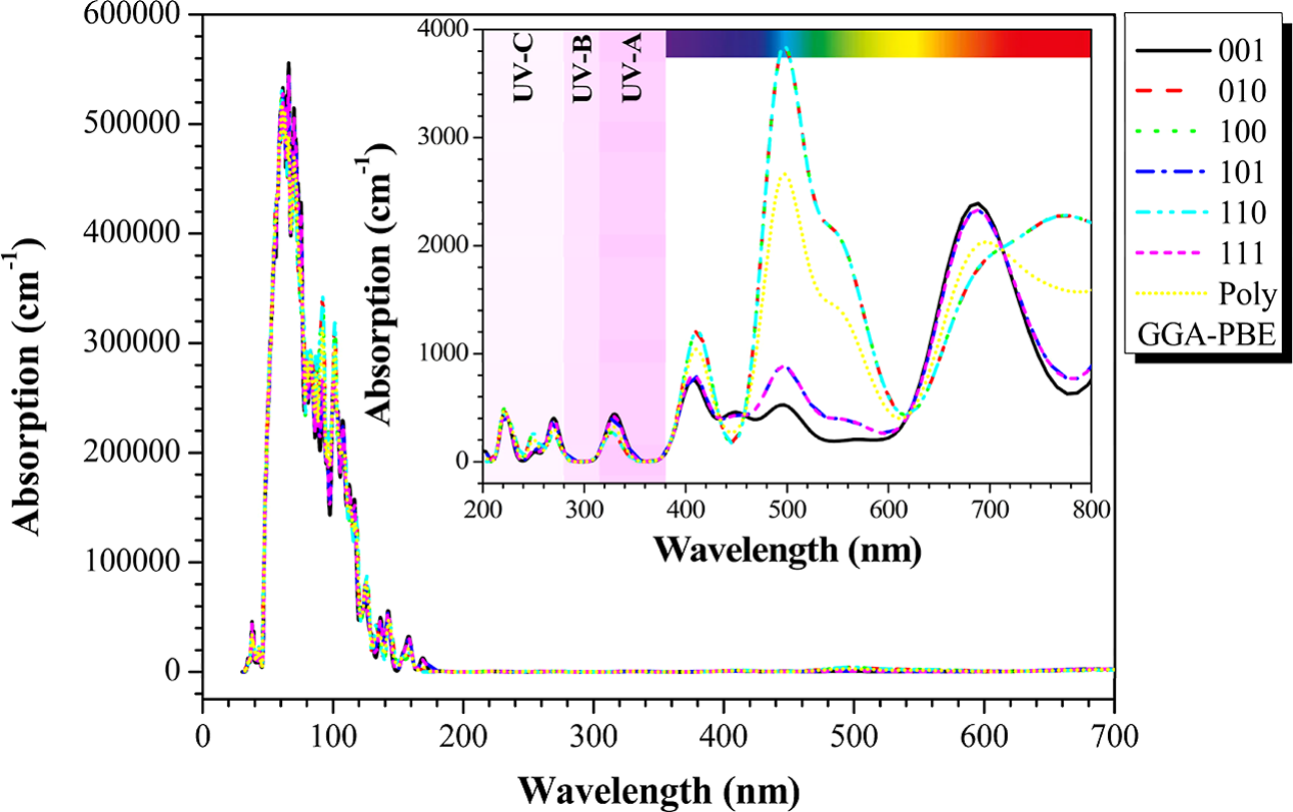
Optical absorption spectra of α-Al_2_O_3_ in the 0–700 nm range, with an inset showing the absorption spectra in the 200–800 nm range, when the incident radiation is polarized along the crystalline planes [001], [010], [100], [101], [110], [111], and for a polycrystalline sample (Poly) using the GGA-PBE approach.

The crystallographic directions [010], [100], [110], and Poly exhibit dominant absorption in the ultraviolet region, which is consistent with the wide-bandgap insulating character of α-Al_2_O_3_. Moderate absorption features extending into the visible range are also observed and may be associated with localized defect states, surface disorder, residual hydrated phases, or nanostructuring effects. Furthermore, between 600 and 700 nm, similar absorption behavior is observed for all crystallographic directions, possibly related to localized optical transitions and structural disorder effects. Therefore, the visible-range absorption should not be interpreted as an intrinsic characteristic of ideal bulk α-Al_2_O_3_. The Poly curve confirms that the material maintains substantial absorption across the entire spectral range analyzed. In contrast, for polarizations in the selected directions, the spectrum shows a significantly amplified response at higher energies (UV region ∼50–150 nm), with potential for selective UV detectors. The optical absorption makes α-Al_2_O_3_ a candidate for heterostructures, where control of crystallographic orientation could adjust the optical response of the device. The strong absorption in the ultraviolet region, combined with the wide bandgap of α-Al_2_O_3_, suggests its suitability for solar-blind UV photodetectors. These devices are particularly relevant for environmental monitoring applications, such as the detection of atmospheric pollutants and combustion processes, contributing to more sustainable industrial and environmental control systems. At the nanoscale, the enhanced surface contribution can lead to increased light-matter interaction, which may improve optical absorption efficiency in the UV region. This behavior is advantageous for the development of nanoscale UV photodetectors and optoelectronic devices with enhanced sensitivity and selectivity.

#### 3.4.2 Reflectivity

When radiation interacts with a crystalline solid, a fraction is transmitted (*T*) through the medium, another is absorbed (*A*) by the material, and a third is reflected (*R*) at the interface, as expressed by the fundamental relationship

T+A+R=1

Thus, reflectivity is directly associated with the visual appearance of the material. The perceived color, for example, results from the spectral distribution of reflected radiation, not from that which was absorbed. Materials with high reflectivity at certain wavelengths therefore exhibit characteristic colors defined by the reflected spectrum.

Figure 7 presents the reflectivity (a.u.) as a function of wavelength (nm) for α-Al_2_O_3_, according to the results obtained by the GGA-PBE approach in various crystallographic directions covering the UV-A, UV-B, UV-C regions and the visible range (inset ∼400–700 nm).

**Figure 7:**
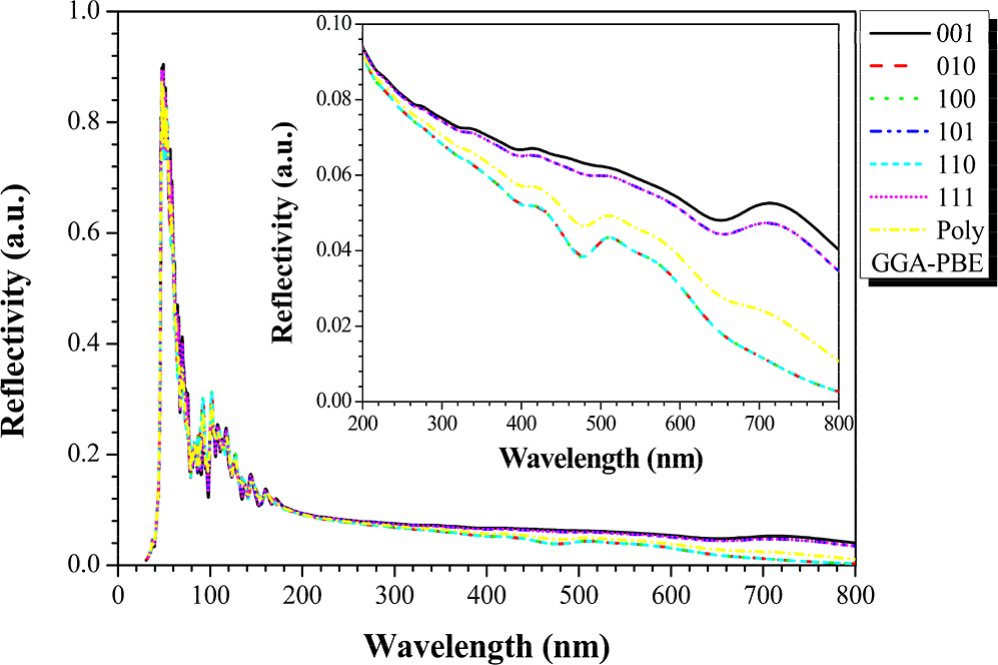
Reflectivity (in arbitrary units) as a function of wavelength (in nanometers) for α-Al_2_O_3_ in the 0–800 nm range, with an inset showing the absorption spectra in the 200–800 nm range, using the GGA-PBE approach, when the incident radiation is polarized along the crystalline planes [001], [010], [100], [101], [110], [111], and for the polycrystalline sample (Poly).

The reflectivity shows similarity between the different crystallographic directions, evidencing little optical anisotropy of the α-Al_2_O_3_ structure. In particular, directions [001], [101], and [111] exhibit higher reflectivity at wavelengths in the visible region, while directions [010], [100], and [110] show a smoother response and, in general, lower reflectivity across most of the analyzed spectrum. Greater reflectivity can be identified in specific regions, especially between 50 and 150 nm, indicating possible electronic transitions or vibrational modes of the lattice that interact with electromagnetic radiation, mainly in the ultraviolet and visible region.

The dependence of reflectivity on crystallographic directions can be exploited in the development of optical sensors/devices that operate in specific spectral ranges, useful for thermal monitoring or gas detection in controlled atmospheres. However, further studies may include measurements at high temperatures and under different atmospheres, simulating actual operating conditions.

### 3.5 Thermodynamic Properties

Figure 8a shows the evolution of the fundamental thermodynamic functions: enthalpy (*H*), Helmholtz free energy (*F*), the term (*T* × *S*), and (b) heat capacity (*C*_v_) as a function of temperature (*T*) for the α-Al_2_O_3_, calculated using density functional perturbation theory (DFPT) formalism,^35,36^ with the GGA-PBE approximation,^25^ presented in the CASTEP code.^22^

**Figure 8:**
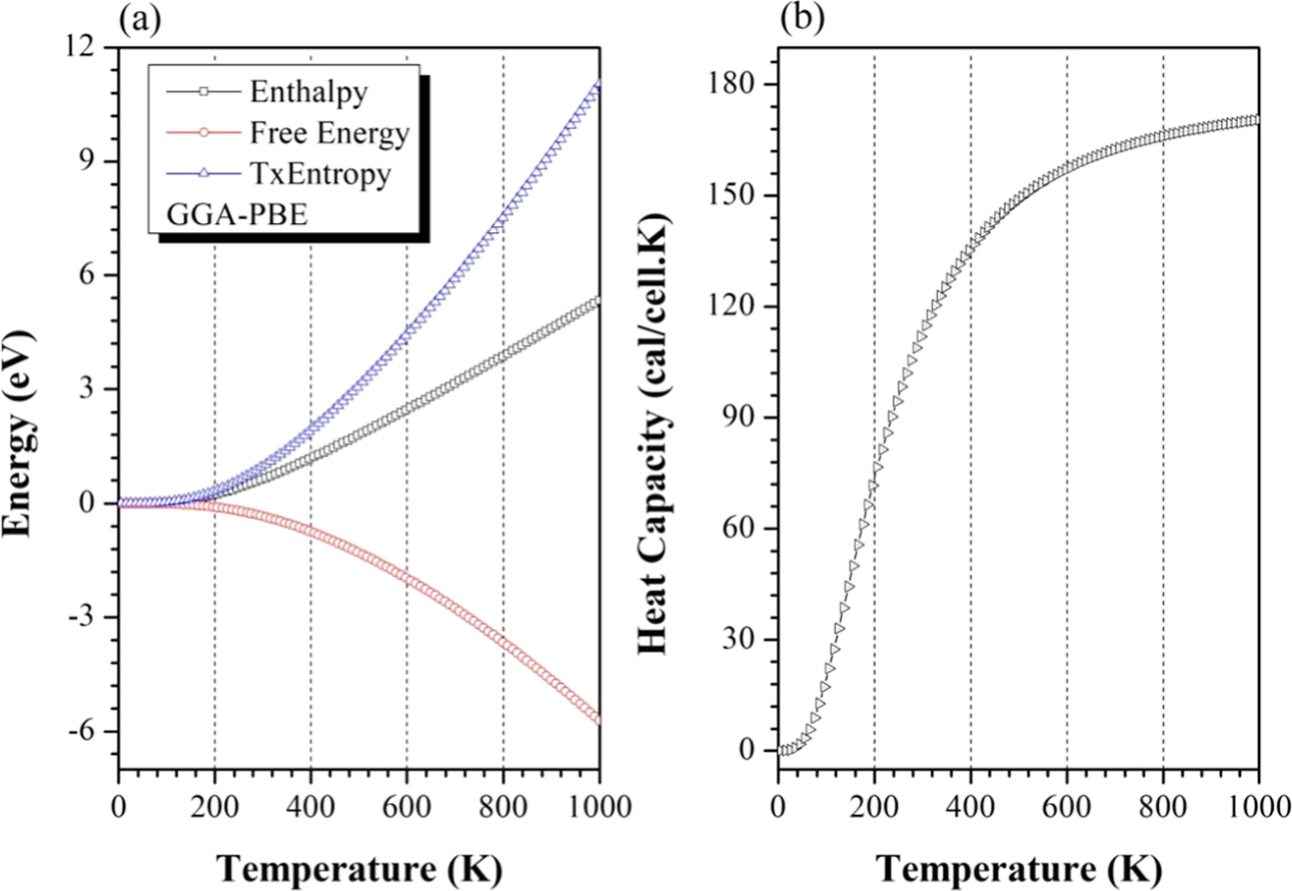
(a) Thermodynamic potentials calculated: enthalpy (black squares), free energy (red circles) and *T* × *S* (blue triangles), and (b) constant volume heat capacity *C*_v_ of trigonal α-Al_2_O_3_, as a function of temperature (in K). (For interpretation of the references to colors in this figure legend, the reader is referred to the web version of this paper.)

The thermodynamic properties were theoretically calculated within the temperature range of 0–1000 K using the quasi-harmonic approximation based on the phonon frequencies obtained from DFPT calculations. Therefore, the temperature-dependent results discussed in this section correspond exclusively to theoretical predictions and do not represent experimental measurements. The *H* curve (black squares—Figure 8a) shows an increasing dependence on temperature (0–1000 K). This behavior is thermodynamically expected and reflects the increase in the internal energy of the crystalline system, mainly associated with the excitation of the vibrational modes of the lattice (phonons). The positive and increasing slope of the curve is directly related to the growth of the material’s heat capacity at constant volume (*C*_v_).

The free energy *F* (red circles—Figure 8a), defined by *F* = *U* – *TS* (where *U* is the internal energy), exhibits a systematic and almost linear decrease as a function of increasing temperature. This decline is dominated by the entropic contribution (–*TS*). The fact that *F* is negative across the entire temperature range investigated (0–1000 K) indicates that the α-Al_2_O_3_ nanostructure is in a thermodynamically stable state and that spontaneous relaxation processes toward equilibrium are favored in this range.

The product of temperature and entropy (*T* × *S*) shows exponential growth (blue triangles—Figure 8a), characteristic of a solid. At low temperatures (close to 0 K), this term approaches zero, in accordance with the third law of thermodynamics. As the temperature rises, the population of excited vibrational states increases, raising the configurational disorder of the system and, consequently, the value of *T* × *S*.

Figure 8b shows *C*_v_ as a function of temperature (K). Growth is initially governed by a law in *T* ^3^ (Debye regime), increasing rapidly as the temperature rises in a range from 0 to 800 K up to the Dulong–Petit limit^47^ for high temperatures. The temperature dependence of the heat capacity (*C*_v_), which approaches a saturation value at high temperatures, reflects the expected lattice vibrational behavior and confirms the reliability of the phonon-based thermodynamic model. Materials exhibiting stable heat capacity profiles are advantageous for thermal management, as they allow for better control of heat flow in electronic, catalytic, and industrial applications. This characteristic is particularly relevant for sustainable technologies, where efficient thermal regulation is essential to minimize energy consumption and enhance the longevity of devices operating under elevated temperatures.

From the computed heat capacity curve, the Debye temperature (θ_D_) of α-Al_2_O_3_ was estimated by fitting the Cv data to the Debye model, yielding a value of approximately 1045 K. This result is in close agreement with experimental values reported for synthetic sapphire, which range from 1040 to 1047 K.^54,55^ The high Debye temperature reflects the strong ionic-covalent bonding and the rigidity of the Al–O network in the corundum structure, and further validates the reliability of the phonon-based thermodynamic calculations performed in this work.

The qualitative and quantitative consistency between the behavior of these calculated functions and that expected for an ionic-covalent crystalline solid, such as α-Al_2_O_3_, validates the DFT-GGA-PBE methodology and the geometric optimization procedure employed. The curves show no discontinuities or abrupt changes in slope in the range studied, suggesting the absence of structural phase transitions to the nanostructure up to 1000 K, which is consistent with the known stability of the trigonal phase in this range. The thermodynamic stability observed, combined with spontaneity (*F* < 0), is a favorable indicator for the use of α-Al_2_O_3_ nanostructures in devices operating under ambient and moderately elevated conditions, such as gas sensors, heterogeneous catalysis, and electrodes for photoelectrochemistry. A detailed understanding of how *H*, *F*, and *S* evolve with *T* is crucial for predicting the performance and durability of these materials under actual operating conditions.

The thermodynamic behavior of α-Al_2_O_3_ demonstrates a consistent and stable response over a wide temperature range, as evidenced by the smooth variation of enthalpy, entropy, and free energy. The decrease in free energy with increasing temperature indicates thermodynamic favorability, confirming the stability of the material under thermal conditions. From an application perspective, this predictable thermodynamic behavior is particularly important for high-temperature processes, where materials are required to maintain structural and functional integrity. In sustainable chemical engineering, such stability contributes to improved energy efficiency and process reliability, reducing energy losses and operational costs in industrial systems. The absence of anomalies in thermodynamic functions confirms the structural stability over the investigated temperature range, which is a critical requirement for high-temperature ceramic applications.

### 3.6 Vibrational Properties of α-Al_2_O_3_

#### 3.6.1 Phonon Spectrum and Cohesive Energy

To assess the dynamic stability of α-Al_2_O_3_, its phonon dispersion relations were computed within the CASTEP package employing the GGA-PBE functional, using the density functional perturbation theory (DFPT) method,^35,36^ within the same convergence criteria presented in the Section 2.2. The resulting spectrum, presented in Figure 9, spans a frequency range of 0–30 THz across the high-symmetry path Γ-A-H-K-Γ-M-L-H in the first Brillouin zone.

**Figure 9:**
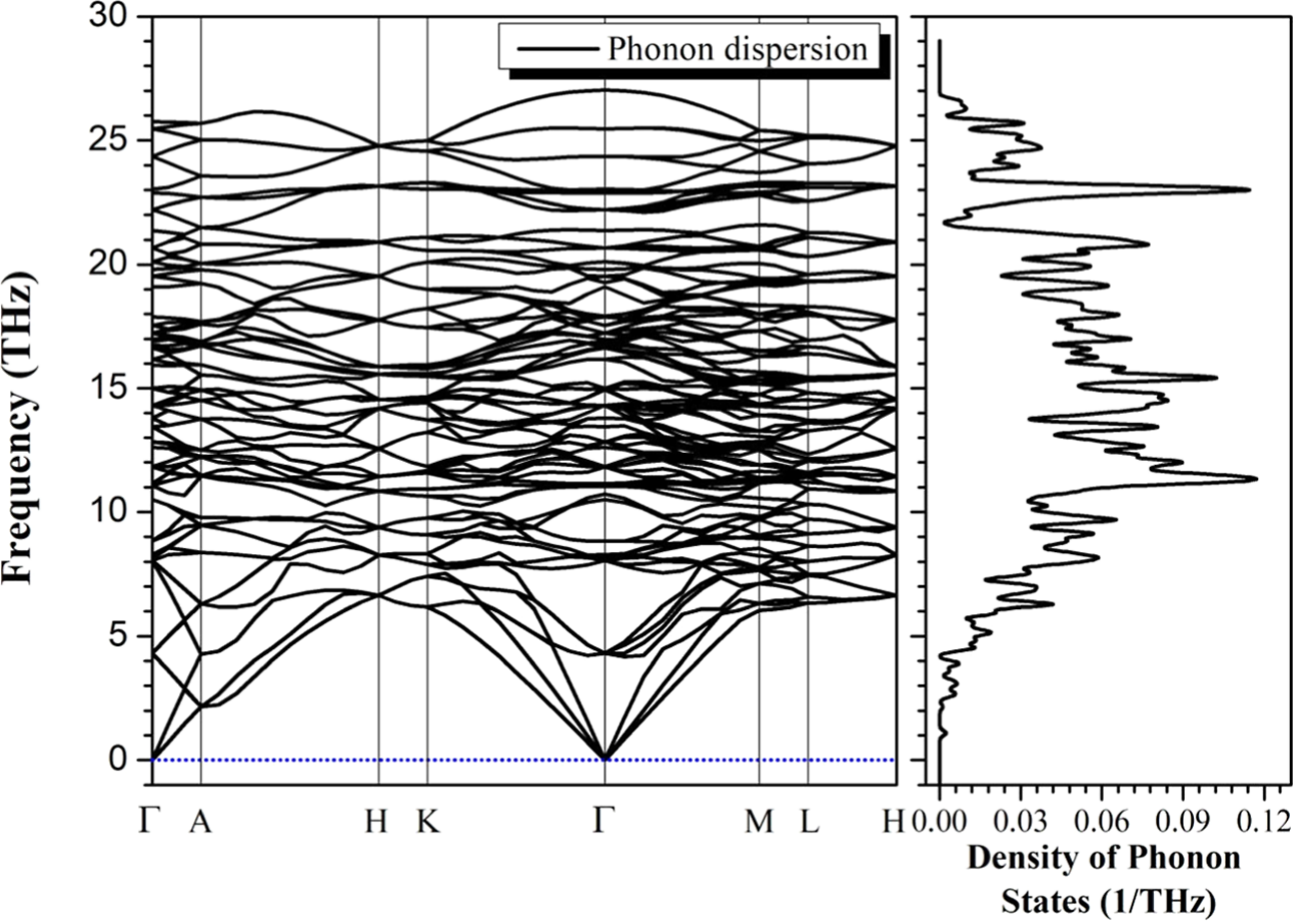
Phonon dispersion curves (left panel) and the density of phonon states (DOS) mode/THz (right panel) of trigonal α-Al_2_O_3_ in the frequency range from 0 to 30 THz, calculated using GGA-PBE exchange-correlation functional.

The absence of imaginary (negative) frequencies throughout the entire Brillouin zone suggests the lattice is dynamically stable, indicating that the crystal structure is robust against lattice distortions. This vibrational stability is particularly important for applications in high-temperature and harsh environments, where structural integrity is essential. In the context of sustainable materials engineering, such stability contributes to long-term durability and reduced material degradation, minimizing maintenance and replacement requirements in industrial processes. The main characteristic of dispersion curves is the presence of two defined frequency regions: a region between 0 and 7.5 THz, within three acoustic modes starting at 0.0 THz which are linear around the Γ point; a second region starting around 5.0 THz are related to the optical modes, with 87 curves in total.

Furthermore, the well-defined phonon density of states suggests efficient lattice vibration behavior, which is directly related to thermal transport properties. Materials with stable and predictable phonon behavior are advantageous for thermal management applications, contributing to energy efficiency in electronic and catalytic systems, which are key aspects of sustainable chemical engineering. At reduced dimensions, phonon behavior can also influence thermal transport and energy dissipation mechanisms. The observed stability of phonon modes suggests that α-Al_2_O_3_ nanostructures may exhibit reliable thermal performance, which is important for nanoscale devices operating under thermal stress. The absence of imaginary phonon modes not only confirms dynamic stability but also supports the reliability of the thermodynamic predictions derived from phonon calculations.

A material achieves stability when it reaches a state of lower energy than the arrangement of its isolated atoms in their most fundamental configuration, forming a cohesive crystal lattice. Thus, the cohesive energy is calculated using the following eq 1:^48,49^

1 $$E_{coh}=\frac{\sum E_{isolated}-E_{bulk}}{N}$$

where *E*_isolated_ is the energies of the isolated Al and O atoms, and *E*_bulk_ is the total energy of the primitive cell. In which by our calculations, *E*_O_^isolated^ = −425.7305 eV is the energy for the O atom, *E*_Al_^isolated^ = −57.0855 eV is the energy for the Al atom, *E*_bulk_ = −8.5096 × 10^3^ eV is the total energy of the primitive cell for the α-Al_2_O_3_ structure, and *N* = 30 is the number of O and Al atoms in the cell. Thus, the cohesive energy obtained for α-Al_2_O_3_ is 5.38 eV per atom. This result shows that the bulk studied has a higher cohesive energy,^50,51^ indicating that the structure presents stability.

#### 3.6.2 Computational Infrared Spectrum

The infrared (IR) spectrum (Figure 10) of the trigonal phase of Al_2_O_3_ (α-Al_2_O_3_, space group *R*3̅*c*) was calculated in the present study using density functional perturbation theory (DFPT). The crystal structure of α-Al_2_O_3_ consists of AlO_6_ octahedra units that share edges, forming a compact hexagonal lattice of oxygen anions with aluminum cations occupying 2/3 of the octahedral sites.

**Figure 10:**
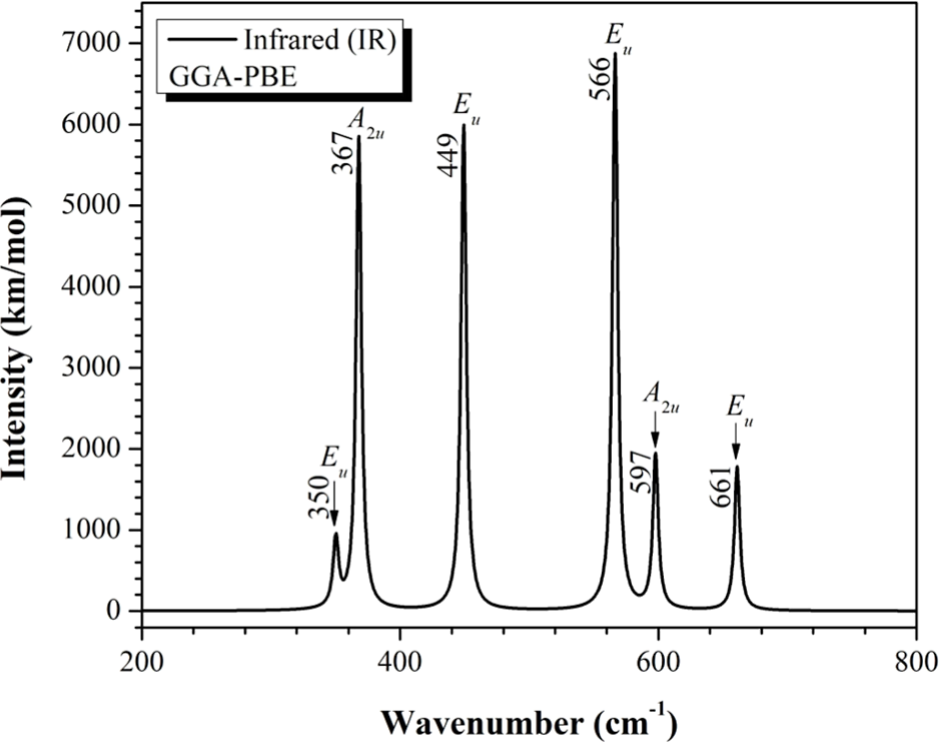
Infrared spectra of α-Al_2_O_3_ in the 200–800 cm^–1^ range, calculated using the GGA-PBE exchange-correlation functional. The numbers correspond to the normal modes.

The theoretical IR spectrum obtained, shown in Figure 10, exhibits active vibrational modes in the infrared range from 200 to 800 cm^–1^. The most intense absorption peak is observed at *E*_u_ = 566 cm^–1^ (569 cm^–1^ ^17^), which is doubly degenerate, reflecting the dynamic distortion of the octahedral lattice, associated with the asymmetric stretching mode of the Al–O bonds within the AlO_6_ octahedra. The second most intense peak appears at *E*_u_ = 449 cm^–1^ (439 cm^–1^ ^17^), doubly degenerate, associated with the O–Al–O bending mode. The third most intense peak at *A*_2u_ = 367 cm^–1^ (397 cm^–1^ ^17^), nondegenerate, corresponds mainly to the O–Al–O bending mode.

The distribution and intensity of the calculated modes are in qualitative agreement with experimental and theoretical spectroscopic data reported for α-Al_2_O_3_ single crystal. Minor discrepancies in peak positions (<20 cm^–1^) are expected due to the harmonic nature of DFT approximations and the known underestimation of vibrational frequencies by GGA-PBE-type functionals, an effect widely documented in calculations of vibrational properties of oxides.^52^

#### 3.6.3 Experimental and Theoretical Raman Spectrum

The crystal structure of the α-Al_2_O_3_ phase belongs to the *R*3̅*c* space group (*D*_3d_ point group), with 30 atoms in the primitive cell (18 oxygen atoms and 12 aluminum atoms). The analysis of the vibrational modes reveals a total of 90° of freedom. These modes are distributed among irreducible representations of the *D*_3d_ group as follows

$$\Gamma_{optic}=5E_{u}+3A_{2u}+10G+10E+3A_{2g}+5E_{g}+2A_{1g}+2A_{1u}$$

These include 87 optical modes, with 3 modes being acoustic (*A*_2u_ + *E*_u_). The selection rules indicate that modes *A*_1g_ and *E*_g_ are active in Raman, while modes *A*_2u_ and *E*_u_ are active in infrared. Therefore, for α-Al_2_O_3_, the following are expected

$$\Gamma_{Raman}=2A_{1g}+2E_{g}$$

$$\Gamma_{IR}=2A_{2u}+4E_{u}$$

Thus, there are 4 active Raman modes and 6 active infrared modes.

Figure 11 shows the experimental (black line) and theoretical (red line) Raman spectra of α-Al_2_O_3_, calculated via DFT using the GGA-PBE functional, in the spectral range from 0 to 1000 cm^–1^. The observed Raman modes are in good agreement with those reported in the literature for the α-Al_2_O_3_ phase.^39,53^ The peaks at 419.8 (417 ^53^) and 646.7 (645 ^53^) cm^–1^ correspond to the *A*_*1g*_ modes predominantly associated with bending and stretching vibrations of the Al–O bonds. The *E*_g_ mode is observed at 751.2 (749 ^53^) cm^–1^, characteristic of the symmetric stretching of the Al–O bond^53^ along the *c* axis. The significant intensity of the peak at 419.8 cm^–1^ in the experimental spectrum is consistent with a well-ordered crystalline structure and the predominance of the trigonal α-Al_2_O_3_ phase. For the calculated Raman (ω_cal_), the absorption peaks are designated to an *A*_1g_ mode at 420.9 and 637.0 cm^–1^ (416 and 640 cm^–1^ ^53^), and the other *E*_g_ modes indicated at frequencies 369.1 and 762.7 cm^–1^ (374 and 774 cm^–1^ ^53^). In nanostructured systems, vibrational modes can be sensitive to size effects, surface disorder, and defects, which may lead to peak shifts and broadening. The good agreement between the experimental and theoretical Raman spectra indicates a high degree of structural ordering within the predominant trigonal α-Al_2_O_3_ phase. However, Raman spectroscopy primarily probes vibrational symmetry and crystalline ordering and does not exclude the possible presence of minor secondary phases, surface disorder, or localized electronic states that may influence the optical absorption behavior discussed in Section 3.2. These results therefore support the structural integrity of the dominant α-Al_2_O_3_ phase while remaining consistent with the optical observations.

**Figure 11:**
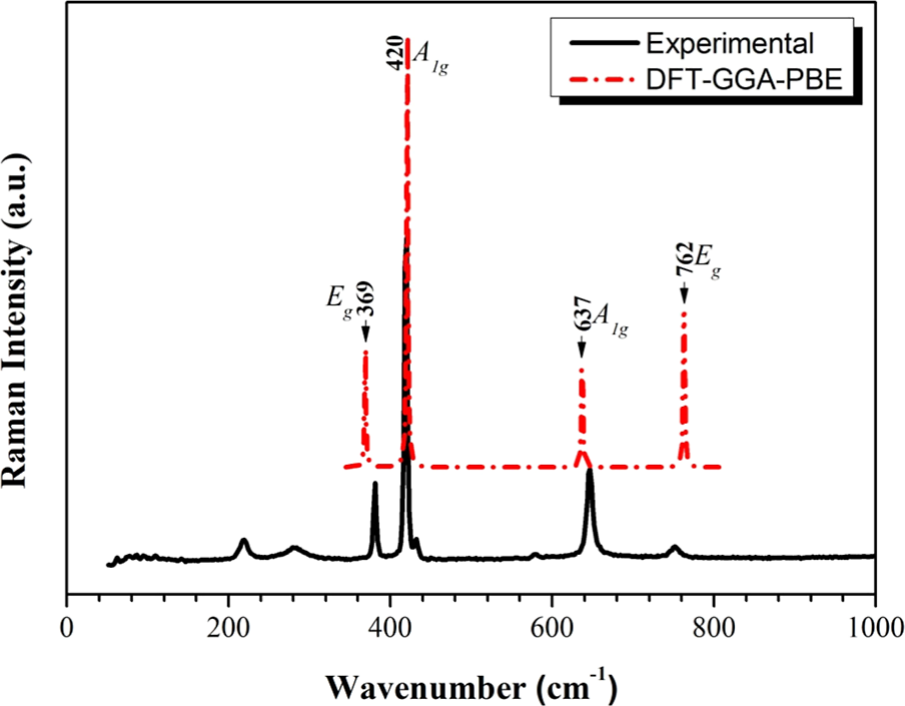
Experimental (black line) and computational (dashed red line) Raman spectra of the α-Al_2_O_3_ phase in the 0–1000 cm^–1^ frequency range. (For interpretation of the references to colors in this figure legend, the reader is referred to the web version of this paper.)

The DFT calculations satisfactorily reproduce the positions and profiles of the experimental modes, although there is a systematic deviation toward slightly higher frequencies, a known feature of harmonic approximations in DFT. The small difference in the number of resolved peaks between the theoretical and experimental spectra can be attributed to the degeneracy of some modes and the resolution of the Raman spectrometer used. Some Raman vibrational modes detected experimentally in the α-Al_2_O_3_ crystal are consistent with those obtained by DFT calculations, demonstrating consistency between both approaches. Table 3 summarizes the observed Raman modes and those calculated theoretically, together with their respective structural assignments.

**Table 3:** Experimental and Calculated Raman-Active Modes of α-Al_2_O_3_, with the Respective Assignments

Raman modes		
α-Al_2_O_3_	α-Al_2_O_3_	modes assignments
ω_obs_ (cm^–1^)	ω_cal_ (cm^–1^)—DFT	
380.8	369.1	*E*_g_
419.8	420.9	*A*_1g_
646.7	637.0	*A*_1g_
751.2	762.7	*E*_g_

Figure 12 shows a correlation graph between theoretical and experimental Raman frequencies (cm^–1^), with a linear fit line whose coefficient of determination is *R* ^2^ = 0.996. The excellent agreement between the calculated and experimental Raman frequencies supports the reliability of the DFPT predictions for the Raman-active modes investigated. However, because only four Raman-active modes are considered, this analysis should be interpreted as a qualitative indication of agreement rather than a statistically robust validation of the theoretical model. Therefore, the *R* ^2^ value is used here only to support the observed correspondence between experimental and calculated peak positions. The scale factor obtained by least-squares adjustment was close to unity, indicating that the GGA-PBE functional provides a reasonably accurate description of the force constants. The residual dispersion of the points around the fit line is quantified by the root-mean-square (RMS) error, which for such a high *R* ^2^ should be relatively low for phonon calculations in oxides and within the typical error margin attributed to harmonic treatment and pseudopotential approximations.

**Figure 12:**
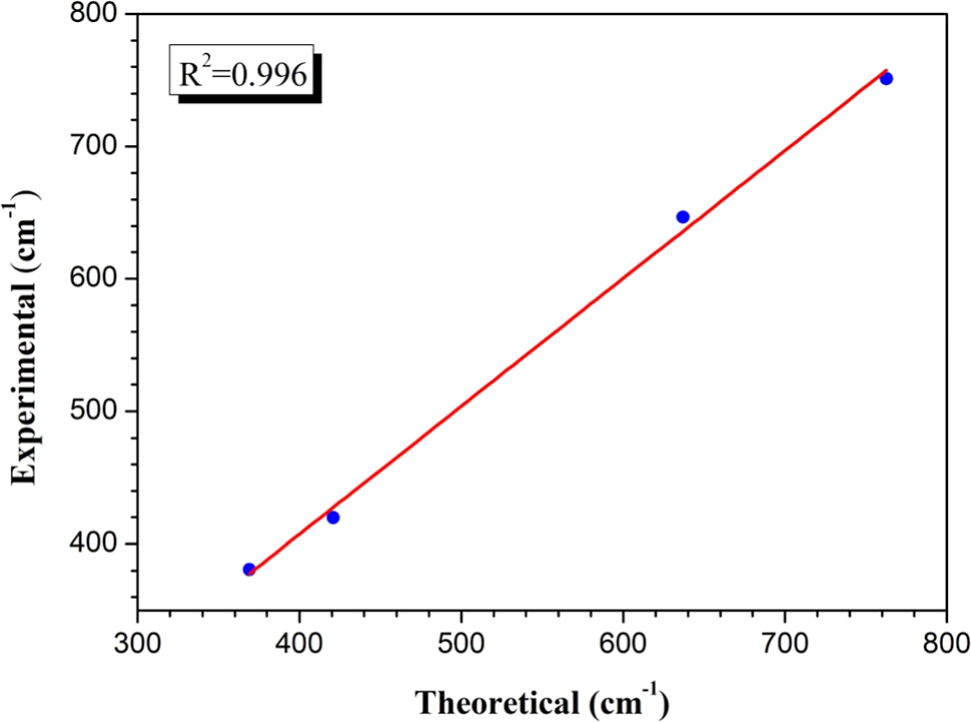
Correlation between theoretical and experimental frequencies (cm^–1^).

### 3.7 Connection to Technological Applications

The optoelectronic, structural, and thermodynamic properties determined in this work bear direct relevance to several established and emerging technological domains. The wide direct bandgap of 6.67–6.68 eV at the Γ-point, combined with the pronounced optical absorption in the deep-ultraviolet region observed in our calculations, supports the use of α-Al_2_O_3_ as a candidate material for solar-blind ultraviolet photodetectors. Such devices, which operate in the wavelength range below 280 nm, are of increasing importance for flame detection, missile warning systems, and environmental monitoring of ozone and pollutants.^56^ The directional dependence of the optical absorption on crystallographic orientation, as demonstrated in our calculated spectra, further suggests that oriented α-Al_2_O_3_ films could be engineered for polarization-selective UV detection. In parallel, the large bandgap and high dielectric constant of α-Al_2_O_3_ have motivated its adoption as a high-k gate dielectric in metal-oxide- semiconductor devices, particularly in GaN-based and SiC-based high-electron-mobility transistors (HEMTs) where thermal stability and large band offsets are required.^41,57^ The electronic band structure and density of states presented in this work, which confirm the O-2p dominated valence band and Al-3s/3p conduction band character, provide fundamental input for modeling the band alignment at α-Al_2_O_3_/semiconductor interfaces used in such devices.

Furthermore, the lattice parameters refined in this study (*a* = 4.75 Å, *c* = 12.99 Å) are directly relevant to the use of α-Al_2_O_3_ (sapphire) as the predominant substrate for GaN-based light-emitting diodes (LEDs). The epitaxial growth of GaN on sapphire, despite the 16% lattice mismatch, underpins the solid-state lighting industry and was central to the development recognized by the 2014 Nobel Prize in Physics.^58,59^ The structural and vibrational data presented here, including the well-resolved Raman modes and the computed phonon dispersion, serve as reference benchmarks for assessing substrate quality, identifying residual phases, and monitoring strain in epitaxial heterostructures. Raman spectroscopy, in particular, is widely employed as a nondestructive tool for phase identification and stress analysis in alumina coatings used as thermal barrier coatings (TBCs) in gas turbine engines, where α-Al_2_O_3_ forms as a thermally grown oxide layer that governs coating adhesion and lifetime.^60^

The thermodynamic stability up to 1000 K demonstrated by the smooth and monotonic evolution of enthalpy, entropy, and free energy, together with the absence of phase transitions, also supports the application of α-Al_2_O_3_ as a protective surface coating for lithium-ion battery cathodes, where thin Al_2_O_3_ layers deposited by atomic layer deposition have been shown to suppress side reactions with the electrolyte and extend cycling life.^4,61^ Additionally, the wide bandgap and the associated deep trap levels in α-Al_2_O_3_ make it one of the most sensitive materials for thermoluminescence (TL) and optically stimulated luminescence (OSL) dosimetry. Carbon-doped α-Al_2_O_3_:C single crystals exhibit a TL sensitivity approximately 50 times higher than that of the standard LiF:Mg,Ti dosimeter, and are currently employed in medical, personal, and environmental radiation dosimetry.^62,63^ The electronic structure characterization reported here provides the fundamental basis for understanding the energy levels involved in these luminescence processes and may guide future doping strategies aimed at optimizing dosimetric performance. These results highlight the potential of α-Al_2_O_3_ as a multifunctional material, where structural, optical, and thermal properties can be simultaneously exploited in advanced ceramic and electronic applications.

The results obtained in this work reveal that the structural stability identified through phonon dispersion and cohesive-energy analysis is directly reflected in the thermodynamic robustness observed up to 1000 K. In parallel, the electronic structure characterized by wide bandgap transitions and dominant O-2p/Al-3s-3p orbital contributions is closely associated with the ultraviolet optical absorption behavior. Furthermore, the consistency between DFPT vibrational calculations and experimental Raman/IR spectra confirms that the local bonding environment and crystal ordering strongly influence both optical and thermodynamic responses. These correlations demonstrate that the structural, vibrational, electronic, and thermodynamic properties of α-Al_2_O_3_ are interconnected rather than independent characteristics.

## 4 Conclusions

This integrated experimental–theoretical study establishes direct relationships between the structural stability, electronic structure, vibrational behavior, optical response, and thermodynamic properties of trigonal α-Al_2_O_3_ nanostructures. Rather than treating these properties independently, the present work demonstrates how lattice stability, local bonding environment, and electronic transitions collectively determine the observed optical and thermodynamic behavior of the material. The reference data and crystal lattice parameters show good agreement. A direct optical bandgap of 5.6 eV was determined by UV–vis spectroscopy; this value is lower than the bulk structure due to surface defects and intragap states, which are common in nanostructured materials. The experimental trends were successfully replicated by DFT calculations using both the LDA-CAPZ and GGA-PBE functionals. An analysis of the electronic structure confirms a direct bandgap at the Γ point (6.67–6.68 eV), with O-2p states predominating at the valence band maximum and Al 3s/3p states forming most of the conduction band minimum. Based on considerable absorption in the UV region and moderate absorption extending into the visible range, the calculated optical absorption and reflectivity spectra reveal moderate anisotropy in the crystallographic directions considered. This directional dependence suggests possible applications in optical devices and polarization-sensitive UV photodetectors. Regarding stability, the phonon dispersion of trigonal α-Al_2_O_3_ does not exhibit a negative (imaginary) phonon frequency, demonstrating that the structure is at a local minimum. Furthermore, through cohesive energy analysis, the system is shown to be energetically stable. The linear correlation coefficient *R* ^2^ = 0.996 between theoretical and experimental frequencies indicates that the analysis of vibrational properties using DFPT calculations was able to reproduce the experimental Raman spectrum with remarkable accuracy. Up to 1000 K, thermodynamic analysis shows that entropy, free energy, and enthalpy change monotonically with temperature; negative free energy suggests potential spontaneous stability over this range. This thermodynamic behavior validates the suitability of α-Al_2_O_3_ for applications under ambient and moderately high temperatures. The results demonstrate that α-Al_2_O_3_ nanostructures exhibit thermodynamic stability under elevated-temperature conditions, well-defined vibrational signatures for phase identification, direct bandgap characteristics suitable for UV optoelectronics, and excellent structural stability. The extensive data set provided here offers a reliable reference when designing alumina-based materials for catalytic supports, protective coatings, optical sensors, and electronic devices where controlled optoelectronic and vibrational responses are required. In addition, the strong thermal and chemical stability highlights its potential as a durable material for catalytic supports and protective coatings. From a materials-performance perspective, the combination of thermal robustness, chemical stability, and predictable thermodynamic behavior positions α-Al_2_O_3_ as a promising material for long-term use in environmental monitoring, high-temperature sensing, and catalytic processes. These characteristics contribute to improved energy efficiency, reduced material degradation, and extended operational lifetimes, which are key factors in sustainable chemical engineering applications. Overall, this work provides a reliable reference for the design and optimization of alumina-based materials with controlled optoelectronic and vibrational properties for advanced and sustainable technological applications. In particular, the UV absorption characteristics and wide bandgap reported here support the application of this material in solar-blind UV photodetection and as a high-k gate dielectric. The refined lattice parameters and vibrational signatures serve as benchmarks for epitaxial growth on sapphire substrates and for phase monitoring in thermal barrier coatings. The thermodynamic stability and electronic structure data further underpin the use of α-Al_2_O_3_ as a protective coating for battery cathodes and as a host matrix for radiation dosimetry. More importantly, this work demonstrates that the integration of experimental and theoretical approaches provides a robust pathway to predict and tailor the multifunctional behavior of ceramic nanomaterials, contributing to the design of next-generation devices.
